# Cadence (steps/min) and intensity during ambulation in 6–20 year olds: the CADENCE-kids study

**DOI:** 10.1186/s12966-018-0651-y

**Published:** 2018-02-26

**Authors:** Catrine Tudor-Locke, John M. Schuna, Ho Han, Elroy J. Aguiar, Sandra Larrivee, Daniel S. Hsia, Scott W. Ducharme, Tiago V. Barreira, William D. Johnson

**Affiliations:** 1 Department of Kinesiology, University of Massachusetts Amherst, 160A Totman Building, Amherst, Massachusetts 01003 USA; 2 Pennington Biomedical Research Center, Baton Rouge, Louisiana, 70808 USA; 3 School of Biological and Population Health Sciences, Oregon State University, Corvallis, Oregon 97331 USA; 4 School of Community Health Sciences, Counseling and Counseling Psychology, Oklahoma State University, Stillwater, OK 74078 USA; 5 School of Education, Syracuse University, Syracuse, NY 13244 USA

**Keywords:** Walking, Physical activity, Pedometer, Accelerometer, Exercise

## Abstract

**Background:**

Steps/day is widely utilized to estimate the total volume of ambulatory activity, but it does not directly reflect intensity, a central tenet of public health guidelines. Cadence (steps/min) represents an overlooked opportunity to describe the intensity of ambulatory activity. We sought to establish thresholds linking directly observed cadence with objectively measured intensity in 6–20 year olds.

**Methods:**

One hundred twenty participants completed multiple 5-min bouts on a treadmill, from 13.4 m/min (0.80 km/h) to 134.0 m/min (8.04 km/h). The protocol was terminated when participants naturally transitioned to running, or if they chose to not continue. Steps were visually counted and intensity was objectively measured using a portable metabolic system. Youth metabolic equivalents (METy) were calculated for 6–17 year olds, with moderate intensity defined as ≥4 and < 6 METy, and vigorous intensity as ≥6 METy. Traditional METs were calculated for 18–20 year olds, with moderate intensity defined as ≥3 and < 6 METs, and vigorous intensity defined as ≥6 METs. Optimal cadence thresholds for moderate and vigorous intensity were identified using segmented random coefficients models and receiver operating characteristic (ROC) curves.

**Result:**

Participants were on average (± SD) aged 13.1 ± 4.3 years, weighed 55.8 ± 22.3 kg, and had a BMI z-score of 0.58 ± 1.21. Moderate intensity thresholds (from regression and ROC analyses) ranged from 128.4 steps/min among 6–8 year olds to 87.3 steps/min among 18–20 year olds. Comparable values for vigorous intensity ranged from 157.7 steps/min among 6–8 year olds to 119.3 steps/min among 18–20 year olds. Considering both regression and ROC approaches, heuristic cadence thresholds (i.e., evidence-based, practical, rounded) ranged from 125 to 90 steps/min for moderate intensity, and 155 to 125 steps/min for vigorous intensity, with higher cadences for younger age groups. Sensitivities and specificities for these heuristic thresholds ranged from 77.8 to 99.0%, indicating fair to excellent classification accuracy.

**Conclusions:**

These heuristic cadence thresholds may be used to prescribe physical activity intensity in public health recommendations. In the research and clinical context, these heuristic cadence thresholds have apparent value for accelerometer-based analytical approaches to determine the intensity of ambulatory activity.

**Electronic supplementary material:**

The online version of this article (10.1186/s12966-018-0651-y) contains supplementary material, which is available to authorized users.

## Background

There is growing interest in translating public health physical activity recommendations using step-based metrics. A step is an intuitively obvious unit of human ambulatory behavior. While steps/day provides important information regarding ambulatory volume, this metric does not directly reflect intensity, an important constituent of public health guidelines. There is increasing consensus [1] that 100 steps/min is a reasonable heuristic (i.e., evidence-based, practical, rounded value) threshold indicative of minimally moderate intensity ambulation (≥ 3 metabolic equivalents [METs]) in adults. However, to date, the evidence supporting cadence thresholds corresponding to moderate and vigorous intensity MET cut points for children, adolescents and young adults remains unclear (to avoid confusion hereafter, we have used the term “thresholds” when referring to cadence values corresponding to MET “cut points”).

It is expected that cadence-intensity thresholds will be somewhat higher in children and decrease throughout adolescence as adult stature and movement patterns are attained. This is likely due, in part, to the shorter stature (i.e., leg length) and associated step length of children, thereby requiring higher cadences to achieve a given speed and therefore intensity. However, this explanation is speculative. Four previous studies have collected cadence data (using accelerometers or pedometers) and indirect measures of intensity (e.g., heart rate, accelerometry) in healthy children/adolescents [2–5], with a fifth study collecting cadence data using an accelerometer and comparing with absolutely-defined intensity (indirect calorimetry; METs) [6]. There is limited evidence, however, regarding the relationship between directly observed cadence (the accepted criterion standard) and absolutely-defined intensity. A single published study by Morgan et al. [7] examined absolutely-defined intensity and direct observation of cadence. However, the age range of participants (9–12 year olds), sample size (*n* = 23), and number of evaluated ambulation speeds (4 walking bouts) limits the external validity and generalizability of their findings. In a review of ambulatory activity in children and adolescents [8], we concluded that “further research is needed to confirm and extend values for directly measured cadences, associated speeds and MET values in young people.”

Therefore, the primary aim of this study was to establish heuristic thresholds linking directly observed cadence with absolutely-defined intensity (METs) during ambulatory activity (i.e., walking/running) across the developmental lifespan of 6–20 years of age. These heuristic cadence thresholds may be used to prescribe physical activity intensity in public health recommendations, shape intensity in intervention and clinical settings, and be used for accelerometer-based analytical approaches to determine the intensity of free-living ambulatory physical activity. Since steps are almost ubiquitously reported by consumer and research-grade physical activity monitors, there is great potential for a valid measure of ambulatory intensity that directly reflects the enacted behavior to be harmoniously applied across a wide variety of validated devices.

## Methods

### Study design and regulatory information

CADENCE-Kids was a laboratory-based cross-sectional study conducted at the Pennington Biomedical Research Center in Baton Rouge, Louisiana, United States. All study procedures were reviewed and approved by the Pennington Biomedical Institutional Review Board. Before participating, informed parental consent and participant assent were obtained for children and adolescents 6–17 years of age. Participants between 18 and 20 years of age provided informed consent.

### Participants

A total of 123 children, adolescents and young adults between 6 and 20 years of age were recruited to participate in the study. To ensure a relatively equal distribution of participants across the evaluated age range of this study, an attempt was made to recruit at least 4 boys and 4 girls from each age-year between 6 and 20 years for a minimum total sample size of at least 120 children, adolescents, and young adults. The study’s age span was designed to effectively capture the age and growth-dependent changes in cadence related to height up to and including attainment of adult stature. Since the intentional focus of CADENCE-Kids was on ambulatory activities, exclusion criteria included those who used wheelchairs or had other impairments that could prevent normal ambulation. Other exclusion criteria were hospitalization for mental illness within the past 5 years, any condition/medication that might affect heart rate or metabolic response to exercise testing or be aggravated by exercise, pregnancy, or presence of a pacemaker or other implanted medical device including metal joint replacements.

### Measures

#### Height measures

The participant’s standing height (without shoes) was measured to the nearest 0.1 cm using a wall-mounted stadiometer (Harpenden model; Holtain Ltd., Crosswell, Crymych, Pembrokeshire, UK) with their head aligned in the Frankfort plane. A stadiometer was also used to measure the sitting height of each participant to the nearest 0.1 cm while seated on a table with legs hanging freely and arms resting on the thighs. Each participant completed two standing height and sitting height measurements, with a third measurement required if the first two measurements were > 0.5 cm apart. The average of the two closest measurements was retained for analysis.

#### Weight

The participant’s weight was measured (without socks and shoes) using a digital scale (Tanita SC-240; Tanita corporation, Tokyo, Japan). Each participant completed two measurements and a third measurement was taken if the first two measurements were > 0.5 kg apart. The average of the two closest measurements was retained for analysis.

#### Derived anthropometric indices

Body mass index (BMI) was calculated as weight divided by height squared (kg/m^2^). Percentiles of BMI and BMI z-scores (BMI_z_) were calculated using reference data from the Centers for Disease Control and Prevention [9]. Calculated BMI percentiles were then used to categorize each participant as underweight (BMI < 5th percentile), normal weight (5th ≤ BMI < 85th percentile), overweight (85th ≤ BMI < 95th percentile), or obese (BMI ≥ 95th percentile). Subischial leg length was calculated as standing height minus sitting height [10].

#### Physical Activity intensity

Respiratory gas concentrations (oxygen consumption [VO_2_] and carbon dioxide production [VCO_2_]) and flow volumes (L/min) during treadmill bouts were measured using a validated portable metabolic system (COSMED K4b2, Rome, Italy; [11]). The device was calibrated according to the manufacturer’s recommendation prior to use.

### Metabolic testing procedures

Participants were required to be in a fasted state (no food or calorie/caffeine containing beverages) for at least 4 h prior to the start of metabolic testing. Following a 25-min seated resting period (which included several sedentary activities not reported on herein [i.e., seated rest, coloring in a book, watching a movie), participants sequentially completed a series of up to 10 five-minute ambulatory treadmill bouts at 0% grade. The first treadmill bout began at 13.4 m/min (0.5 mph) and each subsequent bout increased in speed by 13.4 m/min up to a maximum of 134.0 m/min (5 mph; see Additional File 1, for miles/h and km/h conversions). Treadmill testing was terminated following the first bout when the participant naturally transitioned to running, or if they chose to not continue, reflecting their personal tolerance.

A trained technician visually counted accumulated steps with a hand tally counter during each 5-min bout and a video recording was made of each participant’s lower body movements. Video recordings were referred to in the event that staff-disclosed miscounting or when ambiguous data were identified during post-test processing. For the activities evaluated herein, a “step” was counted anytime a participant raised their foot off the treadmill and subsequently replaced it while supporting their own weight [1, 12, 13] Start and end times of each bout were recorded.

### Data processing and aggregation

Breath-by-breath measurements of absolute and mass-specific VO_2_ (L/min and mL·kg·min^− 1^, respectively) within each minute of collected metabolic data were aggregated (averaged) to produce a minute-by-minute data file for each participant. Youth metabolic equivalents (MET_y_) were calculated as mass-specific VO_2_ divided by resting mass-specific VO_2_ [estimated using the Schofield equation; [14] for participants between 6 and 17 years of age, while traditional metabolic equivalents (METs) were calculated as mass-specific VO_2_ divided by 3.5 mL·kg·min^− 1^ for participants between 18 and 20 years of age. MET_y_ was chosen to quantify the energy cost of activity for the participants between 6 and 17 years of age as published evidence has demonstrated this metric provides balance in attenuating the sex- and age-dependence of energy expenditure estimates in children and adolescents across a range of activities [15, 16]. Moreover, we chose to calculate MET_y_ using estimated resting VO_2_, as opposed to directly measured resting VO_2_, based upon our previous research demonstrating the former outperforms the latter in producing an age-independent metric of metabolic intensity among youth across a range of activities [16]. Traditional METs were used to quantify the energy cost of activity among participants 18–20 years of age to enable comparison with the cadence and intensity relationship in adults. A single MET_y_ or METs value was then calculated for each completed treadmill bout by averaging values from minutes 4 and 5 where steady state was achieved. Steady-state ascertainments during treadmill bouts were evaluated by inspecting breath-by-breath VO_2_ variability corresponding to minutes 4 and 5 from each bout. Those bouts with absolute VO_2_ variability < 10% were deemed steady-state [17]. Bouts not meeting this criterion were excluded from analyses. Observed steps/min was calculated by dividing the total steps visually counted in each activity by 5 min, representing the duration of each bout. Step data were retained only when the participant completed the entire 5-min bout.

Moderate, and vigorous intensity MET_y_ and METs cut points were defined as follows. For participants 6–17 years of age, moderate intensity was defined as ≥4 and < 6 MET_y_, and vigorous intensity was defined as ≥6 MET_y_. For participants 18–20 years of age, moderate intensity was defined as ≥3 and < 6 METs, and vigorous intensity was defined as ≥6 METs. Considerable disagreement exists in regards to selecting MET cut points consistent with various intensities of physical activity among children and adolescents [18, 19]. Although 3 METs has typically been considered indicative of moderate intensity among adults [20], evidence in children and adolescents indicates that brisk walking (≈ 5.6 km/h; [21, 22]), a common indicator of moderate intensity physical activity communicated in public health guidelines [23], elicits absolute physical activity intensities closer to 4 METs than 3 METs [16–18]. As such, for participants 6–17 years of age, 4 MET_y_ was used herein to indicate moderate intensity, consistent with previous accelerometer calibration and validation studies in children and adolescents [17, 21]. Similarly, vigorous intensity was defined > 6 METy and METs, consistent with these previous studies.

### Analytic Sample

Of the 1230 possible treadmill bouts (123 participants * 10 treadmill bouts), 249 treadmill bouts were not completed after the participant terminated testing. Of the remaining 981 treadmill bouts, 129 bouts were excluded due to not meeting our a priori steady-state criteria (i.e., participant failed to complete the entire 5-min bout or absolute VO_2_ variability was > 10%) and 4 bouts from a single participant were excluded due to a malfunction of the portable metabolic system. As a result of the 382 excluded bouts described above, a total of 3 participants from the initial 123 did not have any usable data available for analyses. This resulted in a total of 848 treadmill bouts available for analyses among 120 participants (see Additional File 2 to view/download the final analytical data set and Additional File 3 for the accompanying data dictionary).

### Statistical analyses

All statistical analyses were conducted using R (version 3.3.1; R Foundation for Statistical Computing, Vienna, Austria) and significance was defined as *p* < 0.05. Descriptive statistics (means, ranges, frequencies) were calculated to characterize the sample and graphical techniques were used to visually explore variable distributions (histograms and q-q plots) and the relationship between cadence and physical activity intensity (scatter plots with cadence on the x-axis and METs/METs on y-axis).

#### Preliminary Analyses

**Fig. 1:**
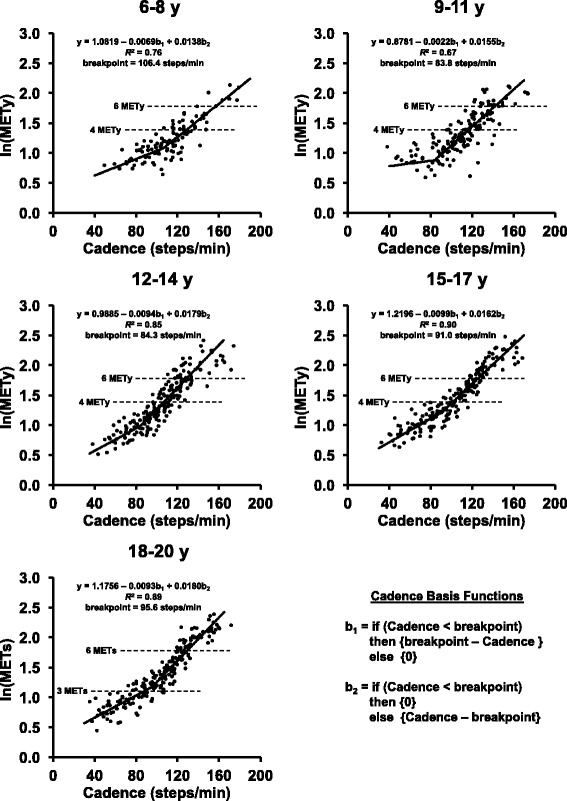
Scatterplots of cadence and ln(MET_y_) for 6–17 year olds, and cadence and ln(METs) for 18–20 year olds. Fitted regression lines and 4 and 6 MET_y_ or 3 and 6 METs intensity levels are superimposed. MET_y_ = youth metabolic equivalents calculated as mass-specific VO_2_ (mL·kg·min^− 1^) divided by resting mass-specific VO_2_ (estimated using the Schofield equation). METs = metabolic equivalents calculated as mass-specific VO_2_ [mL·kg·min^− 1^] divided by 3.5 mL·kg·min^− 1^

Graphical depictions across the range of evaluated treadmill speeds tended to indicate the presence of two distinct linear trends between cadence and metabolic intensity (i.e., a segmented relationship). This was further confirmed by analyses demonstrating higher Marginal *R*^2^ and lower AIC values for segmented models predicting metabolic intensity from cadence than for linear or curvilinear approaches (data not shown). As such, for participants 6–17 years of age, a segmented random coefficients model was fitted with ln(MET_y_) as the dependent variable, and two cadence basis functions (Fig. 1) serving as independent variables. An iterative procedure was used to identify an optimal breakpoint which minimized the model deviance for the segmented model. Utilizing this basic framework, a series of random coefficients models were fitted evaluating the influence of age, biological sex, BMI_z_, and leg length in terms of their potential influence as additional independent variables within predictive models while exploring the significance of their interactions and main effects. For participants 18–20 years of age, the same preliminary analytic strategy was used while using ln(METs) as the dependent variable. ln(MET_y_) and ln(METs) were chosen as the dependent variables in all models to yield homoscedastic residual distributions that more closely approximated normality than those obtained from modelling untransformed MET_y_ or METs.

#### Primary analyses

For participants 6–17 years of age, separate segmented random coefficients models with ln(MET_y_) as the dependent variable, and two cadence basis functions as independent variables (Fig. 1) were fitted for four different age groups (6–8, 9–11, 12–14, and 15–17 years). For participants 18–20 years of age, the same primary analytic strategy was used to fit a single model using ln(METs) as the dependent variable. Marginal *R*^2^ values (i.E., variance explained by each model’s fixed-effects factors) were calculated for each model [24]. Fitted models were used to solve for cadence thresholds corresponding to 4 and 6 MET_y_ for 6–17 year old participants, and 3 and 6 METs for 18–20 year old participants (moderate and vigorous intensity, respectively for MET_y_ and METs). Calibration intervals (99%) for each identified cadence threshold were computed using inverse estimation [25]. Sensitivity and specificity were then quantified relative to each regression-identified cadence threshold. Additionally, receiver operating characteristic (ROC) curves were used to identify optimal cadence thresholds (minimum d = √[(1 – Sensitivity)^2^ + (1 – Specificity)^2^]) that maximized sensitivity and specificity when predicting 4 and 6 MET_y_ (6–17 year olds) or 3 and 6 METs (18–20 year olds) within each age group. Confidence intervals (99%) for optimal thresholds, and area under the curve (AUC) were obtained using the bootstrap with 20,000 replicates. ROC curve AUC values were interpreted as excellent (≥ 0.90), good (0.80–0.89), fair (0.70–0.79), and poor (< 0.70; [26]).

#### Heuristic cadence threshold determinations

Using the more precisely identified regression and ROC curve-based moderate and vigorous intensity cadence thresholds, a more parsimonious set of heuristic thresholds (i.e., evidence-based, practical, rounded values) were generated for use in public health contexts. To this end, heuristic cadence thresholds consistent with moderate and vigorous intensity were defined as the steps/min value (in multiples of 5 steps/min) that minimized the mean distance between the heuristic threshold and the identified regression and ROC curve-based thresholds. Once each heuristic cadence threshold was identified, sensitivity and specificity were quantified.

## Results

Descriptive characteristics of the 120 children, adolescents, and young adults included in the analytic sample are presented in Table 1. As intended, the sample was distributed across sexes and evaluated age groups while the race/ethnic distribution was heterogeneous including large proportions of African-American (35.0%) and Caucasian (62.5%) participants. Mean age of the overall sample was 13.1 ± 4.3 years and the proportion of participants classified as overweight or obese (37.5%) was slightly higher than nationally representative estimates for U.S. 6–19 year olds (≈34%; [27]). Sample sizes, cadence values, VO_2_, MET_y_, and METs for each treadmill bout are available (see Additional File 4 for table of values). We have previously reported descriptive energy expenditure data among this sample in an effort to support the development of the Youth Compendium of Physical Activities [16].

**Table 1:** Descriptive characteristics of the analyzed sample

Variable	Boys (n = 60)			Girls (*n* = 60)			Total (*n* = 120)		
Race/ethnicity (n)									
African-American	20			22			42		
Caucasian	38			37			75		
Other	2			1			3		
Age ranges (n)									
6–8 y	12			10			22		
9–11 y	12			13			25		
12–14 y	12			13			25		
15–17 y	12			12			24		
18–20 y	12			12			24		
Age (y)	13.0	±	4.3	13.1	±	4.2	13.1	±	4.3
Weight (kg)	57.3	±	22.7	54.4	±	22.1	55.8	±	22.3
Height (cm)	157.8	±	19.1	153.0	±	14.1	155.4	±	16.9
Sitting height (cm)	82.1	±	9.5	81.0	±	7.4	81.5	±	8.5
Leg length (cm)	75.7	±	11.3	72.1	±	7.3	73.9	±	9.7
BMI (kg/m^2^)	22.2	±	5.7	22.5	±	6.8	22.4	±	6.3
BMI percentile	64.7	±	31.8	64.4	±	30.6	64.6	±	31.1
BMI z-score	0.61	±	1.25	0.57	±	1.18	0.58	±	1.21
BMI classifications (n)^†^									
Underweight	3			3			6		
Normal weight	33			36			69		
Overweight	11			7			18		
Obese	13			14			27		

*Notes.* Data are presented as frequencies or *M* ± *SD*. BMI = body mass index. ^†^BMI classifications defined as BMI < 5th percentile (underweight), 5th ≤ BMI < 85th percentile (normal weight), 85th ≤ BMI < 95th percentile (overweight), and BMI ≥ 95th percentile (obese)

Preliminary analyses revealed a significant cadence*age interaction (*p* < 0.005) for children and adolescents between 6 and 17 years of age. Further age group stratified analyses revealed no significant sex main effects or cadence*sex interactions among 6–8, 9–11, 12–14, or 15–17 year olds. However, a significant cadence*sex interaction was noted among 18–20 year olds (*p* = 0.036). No significant BMIz main effects or cadence*BMIz interactions were noted for 9–11, 15–17, and 18–20 year olds. However, a significant BMIz main effect was observed among 6–8 year olds (*p* = 0.025) and a significant cadence*BMIz interaction was noted for 12–14 year olds (*p* = 0.020). No significant leg length main effects or cadence*leg length interactions were observed among 6–8 and 15–17 year olds. However, significant cadence*leg length interactions were noted for 9–11 (*p* = 0.033), 12–14 (*p* = 0.002), and 18–20 year olds (*p* = 0.036).

Age-group stratified scatterplots of cadence and ln(MET_y_) for 6–17 year olds, and cadence and ln(METs) for 18–20 year olds, are depicted in Fig. 1. For each age group, graphical displays revealed the presence of a segmented relationship between cadence and metabolic intensity. Estimated cadence thresholds consistent with moderate and vigorous intensity (6–17 year olds: 4 and 6 MET_y_, respectively; 18–20 year olds: 3 and 6 METs, respectively) from the age-group stratified regression models are presented in Table 2. In general, an inverse relationship between cadence thresholds and age was observed. Identified thresholds consistent with moderate intensity varied from a high of 128.4 steps/min among 6–8 year olds to a low of 87.3 steps/min among 18–20 year olds. Regression-based thresholds consistent with vigorous intensity ranged from 157.7 steps/min among 6–8 year olds to 126.3 steps/min among 15–17 year olds. Optimal cadence thresholds consistent with moderate and vigorous intensity identified via ROC curve analyses are presented in Table 3. Similar to regression-based results, optimal cadence thresholds appeared to be inversely related to age. Optimal thresholds consistent with moderate intensity ranged from 121.3 steps/min among 6–8 year olds to 95.9 steps/min among 18–20 year olds. Optimal thresholds consistent with vigorous intensity ranged from 149.0 steps/min among 6–8 year olds to 119.3 steps/min among 18–20 year olds. Overall, AUC from ROC curves indicated that cadence served as an excellent predictor (all AUC ≥ 0.93) of moderate and vigorous intensity activity. Heuristic cadence thresholds consistent with moderate and vigorous intensity are presented in Table 4. To reiterate, these thresholds were identified as the cadence value (in multiples of 5 steps/min) that minimized the mean distance between the heuristic cadence thresholds and the identified regression and ROC curve-based thresholds. These heuristic cadence thresholds ranged from 125 to 90 steps/mins for moderate intensity and 155 to 125 steps/min for vigorous intensity, with higher cadences for younger age groups. Sensitivity and specificity values for these heuristic cadence thresholds were similar to the regression and ROC curve-based thresholds (Tables 2 and 3).

**Table 2:** Identified cadence thresholds (steps/min), sensitivity, and specificity corresponding to moderate and vigorous-intensity derived from regression analyses

		6–8 year olds^a^		9–11 year olds^a^		12–14 year olds^a^		15–17 year olds^a^		18–20 year olds^b^	
Intensity	Measure	Cadence	99% CI^†^	Cadence	99% CI^†^	Cadence	99% CI^†^	Cadence	99% CI^†^	Cadence	99% CI^†^
Moderate	Thr	128.4	121.7–134.5	116.5	109.3–123.9	106.6	102.5–111.0	101.3	97.5–104.8	87.3	79.4–95.7
	Se (%)	60.6		89.1		90.9		95.2		94.2	
	Sp (%)	94.7		89.5		86.7		92.5		80.0	
Vigorous	Thr	157.7	151.8–164.0	142.7	134.6–152.8	129.3	124.1–135.6	126.3	122.9–129.8	129.9	126.3–133.6
	Se (%)	85.7		38.1		66.7		86.5		75.6	
	Sp (%)	100.0		97.7		96.2		98.6		98.1	

*Notes*. Thr = Cadence thresholds in steps/min. Se = sensitivity. Sp = specificity. ^a^ Thresholds estimated using MET_y_ (mass-specific VO_2_ [mL·kg·min^−1^] divided by resting mass-specific VO_2_ [estimated using the Schofield equation]) with moderate and vigorous intensity cut points of 4 and 6 MET_y_, respectively. ^b^ Thresholds estimated using METs (mass-specific VO_2_ [mL·kg·min^− 1^] divided by 3.5 mL·kg·min^− 1^) with moderate and vigorous intensity cut points of 3 and 6 METs, respectively. ^**†**^Calibration intervals (99%) derived using inverse estimation

**Table 3:** Identified cadence thresholds (steps/min), sensitivity, specificity, and area under the curve (AUC) corresponding to moderate and vigorous-intensity derived from ROC curve analyses

		6–8 year olds^a^		9–11 year olds^a^		12–14 year olds^a^		15–17 year olds^a^		18–20 year olds^b^	
Intensity	Measure	Cadence	99% CI	Cadence	99% CI	Cadence	99% CI	Cadence	99% CI	Cadence	99% CI
Moderate	Thr	121.3	106.3–134.9	116.3	112.6–120.1	108.7	100.6–114.4	105.6	97.7–107.5	95.9	85.7–96.4
	Se (%)	84.8		89.1		87.5		91.3		87.8	
	Sp (%)	88.2		89.5		94.3		97.8		96.7	
	AUC	0.94	0.88–0.98	0.94	0.86–0.99	0.97	0.95–0.99	0.99	0.98–1.00	0.97	0.95–0.99
Vigorous	Thr	149.0	147.5–167.2	121.9	121.2–135.8	116.3	109.9–130.5	124.3	117.2–126.3	119.3	118.3–129.0
	Se (%)	100.0		100.0		97.2		92.3		100.0	
	Sp (%)	99.0		77.5		82.2		97.9		87.7	
	AUC	1.00	0.99–1.00	0.93	0.86–0.98	0.96	0.92–0.98	0.99	0.98–1.00	0.98	0.97–0.99

*Notes*. Thr = Cadence thresholds in steps/min. Se = sensitivity. Sp = specificity. AUC = area under the curve. ^a^ Thresholds estimated using MET_y_ (mass-specific VO_2_ [mL·kg·min^−1^] divided by resting mass-specific VO_2_ [estimated using the Schofield equation]) with moderate and vigorous intensity cut points of 4 and 6 MET_y_, respectively. ^b^ Thresholds estimated using METs (mass-specific VO_2_ [mL·kg·min^− 1^] divided by 3.5 mL·kg·min^− 1^) with moderate and vigorous intensity cut points of 3 and 6 METs, respectively

**Table 4:** Heuristic cadence thresholds (steps/min) for moderate and vigorous intensity based on regression and ROC analysis

		Regression thresholds		ROC thresholds		Heuristic thresholds		
Intensity	Age (years)	Cadence	99% CI^†^	Cadence	99% CI	Cadence	Se	Sp
Moderate	6-8^a^	128.4	121.7–134.5	121.3	106.3–134.9	125	78.8	93.4
	9-11^a^	116.5	109.3–123.9	116.3	112.6–120.1	115	89.1	86.0
	12-14^a^	106.6	102.5–111.0	108.7	100.6–114.4	110	86.4	95.2
	15-17^a^	101.3	97.5–104.8	105.6	97.7–107.5	105	91.3	96.8
	18-20^b^	87.3	79.4–95.7	95.9	85.7–96.4	90	91.4	86.7
Vigorous	6-8^a^	157.7	151.8–164.0	149.0	147.5–167.2	155	85.7	99.0
	9-11^a^	142.7	134.6–152.8	121.9	121.2–135.8	130	81.0	87.6
	12-14^a^	129.3	124.1–135.6	116.3	109.9–130.5	125	77.8	93.0
	15-17^a^	126.3	122.9–129.8	124.3	117.2–126.3	125	86.5	97.9
	18-20^b^	129.9	126.3–133.6	119.3	118.3–124.9	125	86.7	94.8

*Notes*. Se = sensitivity. Sp = specificity. ^a^ Thresholds estimated using MET_y_ (mass-specific VO_2_ [mL·kg·min^−1^] divided by resting mass-specific VO_2_ [estimated using the Schofield equation]) with moderate and vigorous intensity cut points of 4 and 6 MET_y_, respectively. ^b^ Thresholds estimated using METs (mass-specific VO_2_ [mL·kg·min^− 1^] divided by 3.5 mL·kg·min^− 1^) with moderate and vigorous intensity cut points of 3 and 6 METs, respectively. ^**†**^Calibration intervals (99%) derived using inverse estimation

## Discussion

Cadence has been linked strongly to intensity in adults, with accumulating evidence consistently supporting a cadence of ≥100 steps/min as a reasonable heuristic threshold (i.e., evidence-based, practical, rounded value) associated with absolutely-defined moderate intensity ambulation [1]. CADENCE-Kids was undertaken primarily to extend this evidence base and ultimately to establish similar heuristic thresholds consistent with moderate and vigorous intensity ambulatory activity in children, adolescents and young adults. Across the developmental span of 6–20 years of age, the data herein lead us to conclude that heuristic cadence thresholds range from 125 to 90 steps/min for moderate intensity, and from 155 to 125 steps/min for vigorous intensity, with higher cadences for younger age groups. These heuristic cadence thresholds are not intended to convey absolute precision of intensity, but are instead intended to be used as guiding values to inform generalized cadence-based physical activity recommendations and/or accelerometer data processing and analysis techniques.

To our knowledge there exists only one study, conducted by Morgan et al. [7], that has attempted to establish cadence thresholds using a criterion standard for steps (direct observation) and objective measurement of absolutely-defined intensity (indirect calorimetry). Their analyses indicated that moderate intensity (4 METs) was associated with 140 steps/min in healthy weight 9–10 year olds and 130 in steps/min 11–12 year olds. For overweight/obese children, moderate intensity was associated with 130 steps/min in 9–10 year olds and 120 steps/min in 11–12 year olds. Discrepancies between the thresholds reported by Morgan et al. and those presented herein may be attributed (in part) to a differing definition of moderate intensity (age-adjusted METs = elicited VO_2_ divided by resting VO_2_), as opposed to the MET_y_ definition employed herein (39). Further, their study contained a relatively small sample size (*N* = 23, with only *n* = 4 actually classified as overweight/obese) and their treadmill protocol was limited to only four speeds.

Despite the minimal number of children and adolescents classified as overweight/obese in their study, Morgan et al. [7] indicated that BMI significantly influenced the relationship between cadence and energy expenditure, and suggested that cadence recommendations consistent with moderate intensity should be specific to a given individual’s obesity status. Although our analyses did reveal significant BMIz-related effects among 6–8 and 12–14 year olds with respect to metabolic intensity, the magnitude of these effects were rather small. Additionally, no significant BMIz-related effects among 9–11, 15–17, and 18–20 year olds were observed. To further investigate this point, we conducted additional follow-up analyses to evaluate the potential magnitude of BMI-related differences in identified cadence thresholds by refitting our age-group specific regression models following stratification for obesity status (non-overweight: BMI < 85th percentile vs. overweight/obese: BMI ≥ 85th percentile). For both moderate and vigorous intensity, the average absolute difference in cadence thresholds between non-overweight and overweight/obese participants across all age groups was 2.7 ± 2.7 and 2.5 ± 2.2 steps/min, respectively. This magnitude of difference is small and calls into question the need for separate BMI-based cadence recommendations. On the other hand, leg length did significantly influence the relationship between cadence and intensity among some age groups (9–11, 12–14, and 18–20 year-olds), consistent with previous published work by Beets et al. among adults [28]. Steps/min thresholds for moderate intensity at minimum and maximum values of leg length varied most among 9–11 year -olds (64.3 cm leg length = 121 steps/min; 83.5 cm leg length = 110 steps/min). Considering the maximum 11 steps/min difference in moderate intensity thresholds herein is substantially smaller than the 26 steps/min difference (85 to 111 steps/min for leg lengths of individuals 1.52 to 1.98 m in height) observed among 20 to 40 year-olds by Beets et al. [28], it would appear that leg length may have a smaller influence on the relationship between cadence and metabolic intensity among children and adolescents than adults. However, such comparisons should be taken with caution considering some of the protocol differences between this investigation and that conducted by Beets et al. (treadmill ambulation vs. over-ground walking, up to 10 stages [13.4 to 134.0 m/min] vs. 5 stages [30 to 90 m/min], among others). Regardless, it may be prudent to correct for leg length in predictive models for scientific research purposes, and possibly clinical applications; however, this does not seem feasible or even appropriate within public health contexts where simplicity of messaging is often a priority [29].

Heuristic thresholds derived from segmented regression and ROC analyses among young adults (18–20 years) herein indicated moderate intensity (3 METs) was best defined using a stepping rate of 90 steps/min. This value falls 10 steps/min below the ubiquitous 100 steps/min recommendation indicative of moderate intensity among adults [1]. However, it should be noted that this single 100 steps/min recommendation is a heuristic value itself representing a range of steps/min thresholds (85 to 115+ steps/min) observed from a series of controlled laboratory studies evaluating the relationship between directly observed cadence and metabolic intensity [28, 30–33]. The variability in this range of thresholds is most likely attributable to differences in participant characteristics (e.g., leg length, height, obesity status, etc.), analytical methods (e.g., simple linear regression, curvilinear models, ROC analyses, etc.), and ambulatory protocols (e.g., treadmill vs. over-ground ambulation, three to six ambulation speeds, etc.). As such, the 90 steps/min value observed herein falls within the range of values encapsulated within the 100 steps/min recommendation. This study provides further evidence that an approximate stepping rate of 100 steps/min is indicative of moderate intensity activity in adults.

We must acknowledge that CADENCE-Kids was a laboratory-based study, executed under controlled conditions, and likely does not reflect enacted free-living ambulatory behavior undertaken in all contexts. Children’s physical activity behaviors are known to be more sporadic and incidental in nature as opposed to rhythmic and continuous [34]. For example, Barreira et al. [35] reported that U.S. children and adolescents spent ≈4 h/day at zero cadence during daily accelerometer wear time, ≈8.9 h/day between 1 and 59 steps/min, ≈22 min at 60–79 steps/min, ≈13 min at 80–99 steps/min, ≈ 9 min at 100–119 steps/min, and ≈ 3 min at cadences ≥120 steps/min. However, it is difficult to ascertain instantaneous intensity from breath-by-breath indirect calorimetry, as a steady state is required for quality data collection and interpretation. As such, the results of this laboratory study are defensible in providing initial heuristic values to guide evaluation of children’s and adolescent’s ambulatory activity, specifically continuous walking and running. A separate analyses of simulated free-living activities (including sedentary behaviors) collected as part of the CADENCE-Kids study is planned to examine step accumulation patterns and intensity during these activities. A further, more focused effort on free-living time-stamped ambulatory behavior is required to determine whether or not more instantaneous patterns and rates of movement are indeed better metrics to track in the context of predicting health outcomes. Despite the limitations outlined above, this study provides the foundational evidence required to support the use of cadence-based thresholds for intensity estimation in children, adolescents, and young adults. This evidence may enhance the utility of consumer and research-grade physical activity monitors that uniformly report steps as an output variable.

Building on the existing empirical literature base, CADENCE-Kids implemented a superior study design that included a broad age range across the developmental age span (the largest sample to date), employed the definitive criterion standard of directly observed steps, used indirect calorimetry for measurement of absolutely-defined intensity, utilized a youth-appropriate MET cut point to establish moderate and vigorous intensity, and applied multiple statistical approaches to generate evidence to inform the selection of heuristic cadence thresholds for moderate and vigorous intensity ambulatory behavior. Segmented random coefficients models and ROC curve analyses were used to evaluate the cadences required to achieve moderate and vigorous intensity. Both methods have strengths and weaknesses. For example, regression models can be disproportionally influenced by extreme values while ROC curves are based only on ranks. Hence, even if there are small differences between the two methods, it is important to remember that our primary aim was to define appropriate heuristic thresholds. There are also some limitations to acknowledge. Firstly, we fully acknowledge natural intra- and inter-individual variability in the cadence-metabolic cost relationship, thereby affecting the individual applicability of heuristic cadence thresholds. Secondly, cadence is an ambulatory indicator and thus does not capture the full repertoire of all possible human movements. Thirdly, the data reported herein were generated in a controlled laboratory setting using an incremental treadmill protocol. As discussed above, instantaneous movement rates, typical in the free-living setting, may convey something completely different in terms of energy expenditure, when compared to rhythmic, continuous, and persistent behavior patterns.

This study opens up a new avenue of research into measurement and modulation of young people’s objectively monitored ambulatory behavior. Clearly at least one ensuing and confirmatory study needed is to manipulate cadence (perhaps by auditory prompt) as guided by these proposed heuristic thresholds and gauge the consequent metabolic response. Another is a more dedicated investigation of how anthropometric factors influence the cadence and intensity relationship, exploring the use of simple clinical measures of stature or leg length, for example, to improve upon the broadly inclusive heuristic thresholds proposed here. Finally, it may be possible to move from heuristic thresholds to those that are more individually calibrated; we have recently piloted the possibility of interpreting children’s free-living accelerometer data based on individualized cadences derived a priori from short-distance walking tests [36].

## Conclusions

Despite the acknowledged limitations outlined above for cadence-based thresholds, we believe that the generation of this additional knowledge, combined with that provided herein and previously reported, will be inherently useful for a broad base of research, clinical, and population-based applications and therefore will also provide an important basis for translating common intensity-related information across these overlapping settings. Imagined applications include physical activity researchers employing covert observation techniques to estimate intensity of ambulatory activity in school playgrounds, physical education teachers leading activities that help children explore their own natural cadences under different conditions, and improvements to consumer and research-grade physical activity monitors to provide real-time cadence outputs to users. These thresholds must be used with caution, however, until they are rigorously cross-validated with other study samples. Although confirmatory research is needed to firmly establish any proposed heuristic cadence threshold, additional intellectual effort is needed to broaden potential applications if such a metric is to be maximally useful.

## Additional files

Additional file 1: Table displaying miles/h and km/h conversions in .pdf format. (PDF 29 kb)

Additional file 2: Table displaying final analytical data set in .xls format. (XLSX 115 kb)

Additional file 3: Data dictionary in .xls format (XLSX 9 kb)

Additional file 4: Table displaying sample sizes, cadence values, VO_2_, MET_y_, and METs for each treadmill bout in .pdf format. (PDF 109 kb)

## Abbreviations

AUC: Area under the curve
BMI: Body mass index (weight divided by height squared)
BMIz: BMI Z-scores
METs: Adult metabolic equivalents (Mass-specific VO_2_ divided by 3.5 mL/kg/min)
METy: Youth metabolic equivalents (Mass-specific VO_2_ divided by resting mass-specific VO_2_)
ROC: Receiver Operating Characteristic
VCO_2_: Breath-by-breath carbon dioxide production (mL/kg/min)
VO_2_: Breath-by-breath oxygen consumption (mL/kg/min)
