# Supplementary material for: Cadence (steps/min) and intensity during ambulation in 6–20 year olds: the CADENCE-kids study
Source: Int J Behav Nutr Phys Act. 2018 Feb 26;15:20. doi: 10.1186/s12966-018-0651-y (PMC5828000; doi:10.1186/s12966-018-0651-y)
Supplement: Supplementary file 1 — Table displaying miles/h and km/h conversions in .pdf format. (PDF 29 kb) [file 12966_2018_651_MOESM1_ESM.pdf]

Additional File 1: Treadmill speeds used in CADENCE-Kids			
**Bout**	**m/min**	**km/h**	**miles/h**
1	13.4	0.80	0.5
2	26.8	1.61	1.0
3	40.2	2.41	1.5
4	53.6	3.22	2.0
5	67.0	4.02	2.5
6	80.4	4.82	3.0
7	93.8	5.63	3.5
8	107.2	6.43	4.0
9	120.6	7.24	4.5
10	134.0	8.04	5.0
