# Supplementary material for: Cadence (steps/min) and intensity during ambulation in 6–20 year olds: the CADENCE-kids study
Source: Int J Behav Nutr Phys Act. 2018 Feb 26;15:20. doi: 10.1186/s12966-018-0651-y (PMC5828000; doi:10.1186/s12966-018-0651-y)
Supplement: Supplementary file 4 — Table displaying sample sizes, cadence values, VO2, METy, and METs for each treadmill bout in .pdf format. (PDF 109 kb) [file 12966_2018_651_MOESM4_ESM.pdf]

Additional File 4: Sample sizes, cadence, VO_2, and MET_y or METs for treadmill bouts									
**Treadmill Speed**	**n**	**Cadence (steps/min)**	**Min-Max**	**VO_2 (L/min)**	**Min-Max**	**VO_2 (mL/kg/min)**	**Min-Max**	**MET_y or METs**	**Min-Max**
**13.4 m/min**									
6-8 y	17	82.5±17.9	49.6-120.6	0.39±0.08	0.27-0.55	13.49±2.34	10.47-17.58	2.45±0.37[a]	1.90-3.08
9-11 y	21	69.5±16.7	38.0-95.8	0.47±0.09	0.35-0.70	10.61±1.82	7.25-14.07	2.41±0.33[a]	1.80-3.13
12-14 y	20	63.7±13.6	38.2-90.6	0.51±0.10	0.38-0.79	8.37±1.81	4.85-12.58	2.22±0.31[a]	1.68-2.78
15-17 y	21	55.0±14.0	32.4-83.2	0.57±0.15	0.35-0.95	8.79±1.17	6.00-10.83	2.37±0.30[a]	1.89-3.07
18-20 y	21	50.8±11.5	35.2-85.4	0.51±0.11	0.31-0.69	7.62±1.17	5.48-9.61	2.18±0.34[b]	1.57-2.75
**26.8 m/min**									
6-8 y	20	94.8±14.5	66.4-123.4	0.42±0.09	0.28-0.63	14.59±2.47	9.46-18.55	2.66±0.37[a]	2.06-3.48
9-11 y	18	88.4±16.6	64.0-117.8	0.55±0.12	0.38-0.86	11.62±2.16	8.11-15.84	2.72±0.43[a]	1.85-3.52
12-14 y	24	78.4±11.7	58.2-101.8	0.59±0.11	0.44-0.84	9.91±2.20	6.20-15.02	2.60±0.38[a]	1.99-3.41
15-17 y	21	70.3±11.5	57.4-95.2	0.68±0.18	0.39-1.10	10.05±1.63	6.16-12.52	2.76±0.35[a]	2.19-3.55
18-20 y	18	67.7±13.2	54.6-106.4	0.62±0.11	0.40-0.76	8.81±1.49	6.21-12.13	2.52±0.43[b]	1.77-3.47
**40.2 m/min**									
6-8 y	18	104.9±14.0	83.0-141.8	0.48±0.10	0.34-0.67	16.65±3.06	11.05-24.36	3.05±0.46[a]	2.41-4.40
9-11 y	18	98.4±11.4	81.8-125.6	0.62±0.14	0.45-0.96	13.15±2.33	9.55-18.05	3.06±0.42[a]	2.32-4.01
12-14 y	23	88.2±8.3	74.4-107.2	0.66±0.12	0.48-0.94	11.07±2.12	7.26-15.58	2.89±0.32[a]	2.33-3.44
15-17 y	23	81.9±8.8	67.6-99.0	0.78±0.22	0.47-1.37	11.70±1.67	7.59-13.59	3.20±0.37[a]	2.63-3.97
18-20 y	21	82.1±11.3	69.4-115.0	0.70±0.14	0.39-0.91	10.20±1.97	7.30-15.87	2.91±0.56[b]	2.09-4.54
**53.6 m/min**									
6-8 y	16	113.6±13.9	80.6-147.2	0.55±0.11	0.37-0.74	18.45±3.43	12.62-24.07	3.41±0.49[a]	2.75-4.18
9-11 y	20	106.1±10.4	87.8-139.4	0.70±0.13	0.52-1.02	15.19±2.48	10.47-20.04	3.47±0.42[a]	2.77-4.46
12-14 y	24	98.3±6.5	87.6-110.8	0.78±0.17	0.55-1.12	12.86±2.29	8.85-17.40	3.39±0.43[a]	2.65-4.61
15-17 y	23	94.1±6.8	79.2-104.4	0.89±0.25	0.52-1.56	13.11±1.90	8.93-15.73	3.62±0.41[a]	2.89-4.46

18-20 y	23	93.3±6.8	82.8-107.8	0.77±0.14	0.46-1.01	11.43±1.66	8.91-14.82	3.26±0.47[b]	2.55-4.24
**67.1 m/min**									
6-8 y	14	122.1±6.3	109.6-133.0	0.62±0.14	0.46-0.90	21.16±3.23	16.42-27.03	3.91±0.52[a]	3.08-4.70
9-11 y	20	117.7±9.7	103.6-141.2	0.79±0.14	0.57-1.09	17.28±2.93	12.64-24.35	4.01±0.62[a]	3.20-5.52
12-14 y	23	107.0±6.4	96.0-121.8	0.91±0.19	0.66-1.29	15.13±2.38	10.62-19.51	4.00±0.43[a]	3.18-5.00
15-17 y	21	105.2±7.4	94.8-124.2	0.99±0.28	0.56-1.81	15.44±1.94	10.46-18.26	4.17±0.51[a]	3.12-4.98
18-20 y	23	103.2±5.7	94.4-114.0	0.87±0.18	0.53-1.15	12.73±1.58	10.27-16.35	3.64±0.45[b]	2.93-4.67
**80.5 m/min**									
6-8 y	10	135.1±12.7	119.4-163.2	0.75±0.22	0.56-1.18	25.20±4.34	18.62-30.74	4.68±0.87[a]	3.69-6.57
9-11 y	21	125.2±8.6	106.6-146.2	0.92±0.18	0.67-1.22	20.54±2.96	15.71-26.96	4.71±0.68[a]	3.55-6.63
12-14 y	23	115.3±6.8	102.4-128.0	1.09±0.22	0.75-1.58	17.42±2.70	12.06-22.66	4.70±0.52[a]	3.65-5.99
15-17 y	22	113.9±5.6	103.2-122.4	1.15±0.32	0.66-2.16	17.65±1.99	14.06-20.73	4.82±0.48[a]	3.65-5.68
18-20 y	21	112.3±4.9	103.0-118.6	1.03±0.18	0.61-1.29	15.05±1.75	11.89-18.05	4.30±0.50[b]	3.40-5.16
**93.9 m/min**									
6-8 y	8	148.1±11.1	134.0-170.0	0.82±0.13	0.67-1.05	30.37±5.71	23.19-38.46	5.40±0.75[a]	4.52-6.74
9-11 y	17	134.6±10.8	120.2-161.4	1.09±0.23	0.82-1.64	25.97±4.60	18.76-36.56	5.81±0.98[a]	4.76-8.13
12-14 y	21	122.7±8.1	107.0-133.6	1.27±0.27	0.94-1.84	21.43±3.28	16.08-28.30	5.59±0.63[a]	4.13-6.83
15-17 y	23	122.5±5.4	113.8-130.6	1.41±0.39	0.85-2.71	21.56±2.48	15.58-25.22	5.88±0.64[a]	4.72-7.01
18-20 y	22	120.9±5.1	109.4-127.4	1.21±0.22	0.76-1.71	18.23±1.88	15.36-22.03	5.21±0.54[b]	4.39-6.29
**107.3 m/min**									
6-8 y	4	161.1±20.4	138.0-179.0	1.03±0.20	0.75-1.17	38.86±9.87	26.16-49.34	6.80±1.32[a]	5.05-8.09
9-11 y	11	145.9±14.5	128.4-173.2	1.23±0.19	0.95-1.53	29.08±2.76	24.06-32.48	6.59±0.69[a]	5.94-7.89
12-14 y	22	136.0±15.2	117.4-171.8	1.54±0.29	1.17-2.17	26.20±5.62	17.21-40.29	6.83±0.95[a]	5.21-9.14
15-17 y	21	133.5±9.2	121.4-164.4	1.75±0.55	1.15-3.46	26.63±4.15	18.89-36.63	7.27±1.11[a]	5.54-9.78
18-20 y	22	130.5±9.5	115.4-152.6	1.53±0.34	0.90-2.17	22.66±3.24	18.24-30.47	6.48±0.92[b]	5.21-8.71

**120.7 m/min**									
6-8 y	2	170.1±0.4	169.8-170.4	1.15±0.22	0.99-1.31	41.68±9.89	34.69-48.67	7.57±1.24[a]	6.69-8.45
9-11 y	4	153.3±17.2	131.8-173.8	1.40±0.13	1.26-1.57	32.24±4.08	28.85-38.15	7.48±0.62[a]	6.80-8.29
12-14 y	11	150.1±16.5	125.2-174.0	1.98±0.48	1.34-2.92	32.30±5.45	27.60-46.47	8.41±1.06[a]	6.49-10.26
15-17 y	17	150.5±12.0	130.2-168.6	2.08±0.62	1.39-3.83	32.27±4.80	24.76-40.45	8.70±1.20[a]	6.87-11.07
18-20 y	18	142.2±11.1	122.2-159.6	1.88±0.40	1.25-2.82	27.75±2.65	24.59-33.41	7.93±0.76[b]	7.03-9.55
**134.1 m/min**									
6-8 y	-	-	-	-	-	-	-	-	-
9-11 y	-	-	-	-	-	-	-	-	-
12-14 y	2	143.6±1.7	142.4-144.8	2.41±0.03	2.39-2.43	44.53±4.36	41.45-47.62	10.74±0.68[a]	10.26-11.22
15-17 y	5	153.1±4.3	149.4-160.4	2.69±0.79	1.95-4.02	37.79±3.18	34.31-41.66	10.25±1.07[a]	9.42-11.97
18-20 y	10	153.8±8.9	138.6-171.6	2.14±0.50	1.47-2.79	31.89±3.60	28.01-38.05	9.11±1.03[b]	8.00-10.87

Notes. Values presented as frequencies and M ± SD. VO_2 = oxygen consumption. [a] METy = youth metabolic equivalents calculated as mass-specific VO_2 (mL/kg/min) divided by resting mass-specific VO_2 (estimated using the Schofield equation). [b] METs = metabolic equivalents calculated as mass-specific VO_2 [mL/kg/min] divided by 3.5 mL/kg/min. Dashes indicate no participants completed a specific activity or metric was not quantified for a given age group.
